# Supplementary material for: Evaluation of the Use of Singleplex and Duplex CerTest VIASURE Real-Time PCR Assays to Detect Common Intestinal Protist Parasites
Source: Diagnostics (Basel). 2024 Feb 1;14(3):319. doi: 10.3390/diagnostics14030319 (PMC10855428; doi:10.3390/diagnostics14030319)
Supplement: Supplementary file 1 [file diagnostics-14-00319-s001.zip › diagnostics-2805204-supplementary.pdf]

Supplementary Materials

Singleplex and duplex PCR protocols used in the primary detection of *Blastocystis* sp., *Cryptosporidium* spp., *D. fragilis*, *E. dispar*, *E. histolytica*, and *G. duodenalis* in clinical samples submitted to the Parasitology Reference and Research Laboratory of the National Centre for Microbiology, Majadahonda (Spain).

Molecular detection of *Blastocystis* sp.

Detection of *Blastocystis* sp. was carried out with a direct PCR, targeting a 600 bp fragment of the small subunit ribosomal RNA (*ssu* rRNA) gene of the parasite (1). Amplification reactions (25 µL) included 5 µL of template DNA, and 0.5 µM of the pan-*Blastocystis* primer pair RD5/BhRDr. Amplification conditions consisted of one step at 95 ºC for 3 min, followed by 30 cycles of 1 min each at 94 ºC, 59 ºC and 72 ºC, before a final extension at 72 ºC for 2 min.

Molecular detection of *Cryptosporidium* spp.

The presence of *Cryptosporidium* spp. was assessed by using a nested-PCR protocol to amplify a 587 bp fragment of the gene codifying the small subunit ribosomal RNA (*ssu* rRNA) of the parasite (2). Amplification reactions (50 µL) included 3 µL of the DNA sample and 0.3 µM of the primer pairs CR-P1/CR-P2 (in the primary reaction) and CR-P3/CPB-DIAGR (in the secondary reaction). Both PCR reactions were carried out as follows: one step of 94 °C for 3 min, followed by 35 cycles of 94 °C for 40 s, 50 °C for 40 s and 72 °C for 1 min, before a final extension of 72 °C for 10 min. Sub-typing of the isolates identified as *C. hominis* or *C. parvum* was attempted for the *gp60* gene, by using the AL-3531/AL-3535 and AL-3532/AL-3534 primer pairs (3).

Molecular detection of *Dientamoeba fragilis*

Detection of *D. fragilis* was carried out by using a direct PCR protocol to amplify a 850 bp fragment of the parasite's *ssu* rRNA gene (4). Amplification reactions (25 µL) included 2 µL of DNA sample and 0.5 µM of the primer pairs DF400/DF1250. Amplification conditions consisted of one step at 94 ºC for 3 min, followed by 30 cycles of 94 °C for 1 min, 57 °C for 90 s and 72 °C for 2 min, before a final extension at 72 ºC for 7 min.

Molecular detection of *Entamoeba dispar* and *Entamoeba histolytica*

Differential diagnosis between non-pathogenic *E. dispar* and pathogenic *E. histolytica* was carried out by a real-time PCR (qPCR), targeting a 172 bp fragment of the parasite's *ssu* rRNA gene (5, 6). Amplification reactions (25 µL) consisted of 3 µL template DNA, 0.5 µM of each primer Ehd-239F and Ehd-88R, 0.4 µM of each specific TaqMan probe, and 12.5 µL of TaqMan Gene Expression Master Mix (Applied Biosystems, Waltham, MA, USA). The amplification protocol consisted of an initial hold step of 2 min at 55 °C and 15 min at 95 °C, followed by 45 cycles of 15 s at 95 °C and 1 min at 60 °C.

Molecular detection and characterization of *Giardia duodenalis*

Detection of *G. duodenalis* DNA detection was accomplished by using a qPCR method, targeting a 62 bp region of the gene codifying the parasite's *ssu* rRNA (7). Amplification reactions (25 µL) consisted of 3 µL of template DNA, 0.5 µM of each primer (Gd-80F and Gd-127R), 0.4 µM of probe, and 12.5 µL of TaqMan® Gene Expression Master Mix (Applied Biosystems). Cycling conditions and data analysis were undertaken in the same way as *E. histolytica* detection (see above).

Giardia duodenalis isolates that qPCR testing confirmed as positive were subsequently re-assessed by sequence-based multi-locus genotyping of the genes, with the encoding of the glutamate dehydrogenase (*gdh*) (8), β-giardin (*bg*) (9), and triose phosphate (*tpi*) (10) parasite proteins . We conducted amplifications by applying semi-nested and nested PCR protocols that used the specific primer pairs described in references 8–10.

REFERENCES

1. Scicluna SM, Tawari B, Clark CG. 2006. DNA barcoding of *Blastocystis*. Protist 157:77–85. https://doi.org/10.1016/j.protis.2005.12.001
2. Tiangtip R, Jongwutiwes S. 2002. Molecular analysis of *Cryptosporidium* species isolated from HIV-infected patients in Thailand. Trop Med Int Health 7:357–364. https://doi.org/10.1046/j.1365-3156.2002.00855.x
3. Feltus DC, Giddings CW, Schneck BL, Monson T, Warshauer D, McEvoy JM. 2006. Evidence supporting zoonotic transmission of *Cryptosporidium* spp. in Wisconsin. J Clin Microbiol 44:4303–4308. https://doi.org/10.1128/JCM.01067-0
4. Stark D, Beebe N, Marriott D, Ellis J, Harkness J. 2005. Detection of *Dientamoeba fragilis* in fresh stool specimens using PCR. Int J Parasitol 35:57–62. https://doi.org/10.1016/j.ijpara.2004.09.003
5. Gutiérrez-Cisneros MJ, Cogollos R, López-Vélez R, Martín-Rabadán P, Martínez-Ruiz R, Subirats M, Merino FJ, Fuentes I. 2010. Application of real-time PCR for the differentiation of *Entamoeba histolytica* and *E. dispar* in cyst-positive faecal samples from 130 immigrants living in Spain. Ann Trop Med Parasitol 104:145–149. https://doi.org/10.1179/136485910X12607012373759

6. Verweij JJ, Oostvogel F, Brienen EA, Nang-Beifubah A, Ziem J, Polderman AM. 2003. Prevalence of *Entamoeba histolytica* and *Entamoeba dispar* in northern Ghana. Trop Med Int Health 8:1153–1156. https://doi.org/10.1046/j.1360-2276.2003.01145.x
7. Verweij JJ, Schinkel J, Laeijendecker D, van Rooyen MA, van Lieshout L, Polderman AM. 2003. Real-time PCR for the detection of *Giardia lamblia*. Mol Cell Probes 17:223–225. https://doi.org/10.1016/S0890-8508(03)00057-4
8. Read CM, Monis PT, Thompson RC. 2004. Discrimination of all genotypes of *Giardia duodenalis* at the glutamate dehydrogenase locus using PCR-RFLP. Infect Genet Evol 4:125–130. https://doi.org/10.1016/j.meegid.2004.02.001
9. Lalle M, Pozio E, Capelli G, Bruschi F, Crotti D, Cacciò SM. 2005. Genetic heterogeneity at the beta-giardin locus among human and animal isolates of *Giardia duodenalis* and identification of potentially zoonotic subgenotypes. Int J Parasitol 35:207–213. https://doi.org/10.1016/j.ijpara.2004.10.022
10. Sulaiman IM, Fayer R, Bern C, Gilman RH, Trout JM, Schantz PM, Das P, Lal AA, Xiao L. 2003. Triosephosphate isomerase gene characterization and potential zoonotic transmission of *Giardia duodenalis*. Emerg Infect Dis 9:1444–1452. https://doi.org/10.3201/eid0911.030084
